# Magnetic Resonance Imaging-Visible Perivascular Spaces in the Basal Ganglia Are Associated With the Diabetic Retinopathy Stage and Cognitive Decline in Patients With Type 2 Diabetes

**DOI:** 10.3389/fnagi.2021.666495

**Published:** 2021-11-12

**Authors:** Eun Young Choi, Yae Won Park, Minyoung Lee, Min Kim, Christopher Seungkyu Lee, Sung Soo Ahn, Jinna Kim, Seung-Koo Lee

**Affiliations:** ^1^Department of Ophthalmology, Yonsei University College of Medicine, Seoul, South Korea; ^2^Department of Radiology, Center for Clinical Imaging Data Science, Research Institute of Radiological Science, Yonsei University College of Medicine, Seoul, South Korea; ^3^Department of Endocrinology, Yonsei University College of Medicine, Seoul, South Korea

**Keywords:** basal ganglia, cerebral small vessel disease, cognitive decline, diabetes mellitus, diabetic retinopathy, ganglion cell layer, perivascular space

## Abstract

**Purpose:** The aim of this study was to evaluate whether perivascular space (PVS) severity and retinal ganglion cell layer (GCL) thickness differed based on the stage of diabetic retinopathy (DR) and the cognitive status in patients with DR.

**Methods:** A total of 81 patients with DR (51 in the non-proliferative group and 30 in the proliferative group) were included in this retrospective, cross-sectional study. PVS severity was assessed in the basal ganglia (BG) and centrum semiovale using MRI. The total cerebral small vessel disease (SVD) score was determined based on the numbers of lacunes and microbleeds and the severity of white matter hyperintensity. Optical coherence tomography was used to measure foveal and perifoveal GCL thicknesses. Cerebral SVD markers and cognitive function were compared between the groups, and correlations between the BG-PVS severity and the Mini-Mental Status Examination (MMSE) scores and GCL parameters were evaluated.

**Results:** Patients with proliferative DR had higher BG-PVS severity (*P* = 0.012), higher total cerebral SVD scores (*P* = 0.035), reduced GCL thicknesses in the inferior (*P* = 0.027), superior (*P* = 0.046), and temporal (*P* = 0.038) subfields compared to patients with non-proliferative DR. In addition, the BG-PVS severity was negatively correlated with the MMSE score (*P* = 0.007), and the GCL thickness was negatively correlated with the BG-PVS severity (*P*-values < 0.05 for inferior, superior, and temporal subfields).

**Conclusion:** BG-PVS severity and retinal GCL thickness may represent novel imaging biomarkers reflecting the stage of DR and cognitive decline in diabetic patients. Furthermore, these results suggest a possible link between cerebral and retinal neurodegeneration at the clinical level.

## Introduction

Diabetic retinopathy (DR) is a major microvascular complication of type 2 diabetes mellitus (T2DM) and a predictor of other end-organ complications (Aminian et al., 2020), with a global prevalence of 27% in patients with T2DM (Thomas et al., 2019). DR can be divided into two major categories, namely, non-proliferative DR in the early stages and proliferative DR in the more advanced stages. While pathological preretinal neovascularization is a hallmark feature of proliferative DR (Stitt et al., 2016), retinal neurodegeneration is reported to occur earlier in DR pathogenesis (Sohn et al., 2016). Retinal ganglion and amacrine cells are believed to be affected first by diabetes-induced apoptosis (Ng et al., 2016), which can be identified clinically by a decrease in the ganglion cell layer (GCL) thickness on optical coherence tomography (OCT) imaging (Ng et al., 2016). Moreover, previous studies have shown that patients with DR have an increased risk of cognitive impairment (Crosby-Nwaobi et al., 2012; Gupta et al., 2019), although the pathophysiological mechanisms underlying this process are not well known.

While the retina is the classic target organ for diabetic microangiopathy, the brain has also recently been identified as another target organ for diabetic microvascular complications (Woerdeman et al., 2014). The retina and the brain share similar anatomical and physiological characteristics, as both are embryologically derived from the same tissue and possess similar structural and functional features, such as the blood-retinal/blood-brain barriers and glial cell connections (London et al., 2013). In addition, similar to the retinal microvasculature, an increasing body of evidence indicates that hyperglycemia can induce dysfunction in the cerebral microvasculature, causing pathological neurovascular remodeling, blood-brain barrier disruption, and cell damage to neurons and glial cells (Ergul et al., 2012). The retinal glymphatic system, a network between the superficial capillary plexus and the inner nuclear layers, has also been reported to reflect the state of neurodegenerative and inflammatory disorders in the brain (Petzold, 2016).

Cerebral small vessel disease (SVD) encompasses a wide spectrum of cerebrovascular diseases with similar clinical manifestations. Using MRI, conventional markers for SVD include lacunes, microbleeds, and white matter hyperintensity (WMH) (Chen et al., 2019). Cerebral perivascular spaces (PVS), also known as Virchow-Robin spaces, are pial-lined, interstitial, fluid-filled spaces surrounding the penetrating vessels. A growing body of evidence suggests that enlarged PVS represent a novel biomarker for cerebral SVD (Duperron et al., 2018). Enlarged PVS have been correlated with cerebrovascular disease (Hurford et al., 2014; Potter et al., 2015) and neurodegenerative diseases such as dementia (Ramirez et al., 2016; Banerjee et al., 2017; Sepehrband et al., 2021). Moreover, PVS have been shown to be a component of the glymphatic system, a recently discovered, macroscopic, waste-clearance system in the brain (Jessen et al., 2015). A recent study has demonstrated the glymphatic system damage in T2DM rat models (Jiang et al., 2017), and the enlarged PVS may reflect this damage (Jessen et al., 2015). However, the relationships between cerebral PVS enlargement, retinal neurodegeneration, DR stage, and cognitive decline have not yet been evaluated. Therefore, the comprehensive assessments of the brain and retinal changes should be performed to elucidate the underlying mechanisms for these processes at the clinical level and to acquire practical insights into the design of appropriate treatments for the prevention of cognitive impairment in patients with DR.

Therefore, in this study, we investigated whether MRI-visible PVS, other cerebral SVD markers, and retinal GCL thickness differed based on the DR stage and the cognitive status of patients with DR.

## Materials and Methods

### Patient Population

This retrospective study was approved by the Severance Institutional Review Board, and a waiver of informed consent was obtained (IRB approval number: 2020-3812-001). In total, 158 patients with DR who had undergone detailed neurological examinations for the assessment of cognitive impairment from February 2010 to April 2020 were identified. Among them, 130 patients with DR who had undergone both brain MRI and neuropsychological testing within 1 year of ophthalmologic examinations were enrolled. In this prospective study, participants were excluded based on the following criteria: (1) presence of other retinal diseases (e.g., age-related macular degeneration, central serous chorioretinopathy, retinal vascular occlusion, epiretinal membrane, and macular hole) or optic nerve diseases (e.g., optic neuritis, glaucoma, and ischemic optic neuropathy) (*n* = 19); (2) presence of old infarcts due to large vessel disease or cardioembolic strokes, past intracerebral hemorrhages, or post-traumatic encephalomalacia detected on MRI (*n* = 17); (3) a previous history of intravitreal or sub-Tenon’s injections, vitrectomy surgery, or laser photocoagulation for the treatment of diabetic macular edema (*n* = 7); or (4) a current or past history of severe dementia, other neurological or neurodevelopmental disorders, substance-related problems, or head trauma with loss of consciousness (*n* = 6). After the consideration of these exclusion criteria, a total of 81 consecutive patients with DR were included in the final study population (mean age = 67.3 ± 9.9 years; 49 females and 32 males).

Demographic and clinical characteristics of patients, such as age, sex, vascular risk factors (e.g., hypertension, dyslipidemia, or cardiovascular diseases), and current or remote history of smoking, were collected. The Mini-Mental Status Examination (MMSE) (Han et al., 2008) assessed a range of elements that include time orientation, spatial orientation, memory registration, attention and calculation, memory recall, language, and space-time configuration. The scores of the MMSE test result were divided into normal and abnormal in 15 percentile standards by using the age and education criteria. “Cognitive impairment” was defined as an adjusted MMSE score in the range of 10–23, with less than 10 for “severe dementia” (Creavin et al., 2016). The Seoul Neuropsychological Screening Battery (SNSB) test results were evaluated to determine four cognitive statuses (i.e., memory, language skills, space-time functions, and executive function) and provided percentile score adjusted for age, sex, and education (Ahn et al., 2010). The SNSB test incorporated the MMSE, Clinical Dementia Rating (Juva et al., 1994), and Global Deterioration Scale (Reisberg et al., 1982).

### Assessment of Diabetic Retinopathy Stage and Retinal Ganglion Cell Layer Thickness

Images from laser-scanning ophthalmoscopy (Optos Plc., Dunfermline, United Kingdom) and fluorescein angiography (Heidelberg Engineering, Heidelberg, Germany) were reviewed to diagnose DR and to determine the DR stage. Non-proliferative vs. proliferative DR stages were identified based on the presence of preretinal angiogenesis using fundus fluorescein angiography (Group, 1991). The examination of the GCL thickness in the macula was performed using spectral-domain OCT (Spectralis^®^, Heidelberg Engineering, Heidelberg, Germany), and the retinal-segmentation software accompanying this device was used to identify and quantify the average thickness of each retinal neuronal layer (Reisberg et al., 1982). The GCL failed was defined as the layer between the ganglion cell-inner plexiform layer (Mwanza et al., 2011), and the GCL thickness was measured in the foveal area (a 1-mm zone centered on the fovea) and in the inferior, superior, nasal, and temporal subfields (a 1- to 3-mm perifoveal zone), as defined by the Early Treatment DR Study. The mean GCL thickness of each subfield in both eyes was analyzed. However, if one eye met exclusion criteria, only GCL parameters in the opposite eye were analyzed. Two trained retinal ophthalmologists (with 8 and 18 years of experience, respectively) measured OCT findings, and the averaged values were used for statistical analyses.

### MRI Protocol

All scans were acquired with a 3.0-T scanner (Achieva; Philips Healthcare, Best, Netherlands) with a 32-channel head coil. Head motion was minimized with restraining foam pads. The MRI imaging protocol included T2-weighted (repetition time [TR]/echo time [TE]: 2,800–3,000/80–100 ms; field of view (FOV): 230–240 mm; section thickness: 5 mm; matrix: 256 × 256), T2*-weighted gradient-echo images (TR/TE: 500–1,000/15–25 ms; section thickness: 5 mm; matrix: 256 × 256), and fluid-attenuated inversion recovery (FLAIR) images (TR/TE: 9,000–10,000/110–125 ms; FOV: 240 mm; section thickness: 5 mm; matrix: 256 × 256).

### Assessment of Cerebral Small Vessel Disease Markers

The PVS was identified and rated on axial, T2-weighted, MR images based on the previously reported criteria (Wardlaw et al., 2013) by two trained neuroradiologists (with 15 and 8 years of experience, respectively) who were blinded to clinical information. A 4-point visual rating scale (0 = absent PVS, 1 = less than 10 PVS, 2 = 11–20 PVS, 3 = 21–40 PVS, and 4 = more than 40 PVS) was applied to the basal ganglia (BG) and the centrum semiovale (CSO) (Wardlaw et al., 2013). The number of MRI-visible PVS was counted in each hemisphere, and the highest score was recorded. The final consensus rating scale results were used for the analysis.

Lacunes were defined as lesions larger than 3 mm and less than 15 mm in the subcortical area with high signal intensity on the T2-weighted images and a perilesional halo on FLAIR imaging (Wardlaw et al., 2013). The number of lacunes was manually counted in the brain stem, BG, thalamus, pons, and cerebral white matter. Microbleeds were defined as being 10 mm or less in diameter on axial sections of the T2-weighted gradient-echo images, using the previously reported criteria (Greenberg et al., 2009). The total number of microbleeds was counted.

The WMH was defined as a hyperintense white matter lesion on FLAIR images based on the STRIVE (STandards for ReportIng Vascular changes on nEuroimaging) criteria (Wardlaw et al., 2013) and was graded according to the Fazekas scale as deep WMH (0 = absent, 1 = punctate, 2 = early confluent, and 3 = confluent) or periventricular WMH (0 = absent, 1 = caps or pencil-thin lining, 2 = smooth halo, and 3 = irregular WMH extending into the deep white matter) (Fazekas et al., 1987; Wardlaw et al., 2013). The total Fazekas score was calculated by adding together the periventricular and deep WMH scores (0–6) (Park et al., 2018).

The total cerebral SVD score was calculated for each patient using an ordinal scale ranging from 0 to 4 by counting the presence of each of the four MRI features of cerebral SVD, with a score of 1 point indicating the presence of high BG-PVS severity (a score ≥ 2), lacunes, microbleeds, and severe WMH (total Fazekas score > 3) (Staals et al., 2014). Lacunes, microbleeds, and WMHs were rated through consensus discussions between the two trained neuroradiologists.

### Statistical Analysis

Baseline clinical and imaging characteristics were compared between non-proliferative DR and proliferative DR groups using the chi-squared or Fisher’s exact test for categorical variables or the independent *t*-test or Mann–Whitney *U* test for continuous variables, according to normality. Interobserver agreements for imaging evaluations were calculated using the Cohen’s kappa index. In addition, correlations between the BG-PVS severity and the total cerebral SVD score with the MMSE score and GCL parameters were evaluated using the Spearman’s correlation coefficient analysis. The multivariable logistic regression analysis using the enter method was performed to determine the effect of selected cerebro-ophthalmic parameters (with *P*-values of < 0.10 in previous comparison and correlation tests) on the final outcome, i.e., DR progression to the proliferative stage. An identical process was performed to predict cognitive impairment (adjusted score of < 24 on the MMSE).

All statistical analyses were performed using the Statistical Package for the Social Sciences (SPSS/IBM Corporation; Chicago, IL, United States) version 23.0 software for Windows. A *P*-value < 0.05 was considered statistically significant.

## Results

Among 81 patients, 51 patients were classified as having non-proliferative DR and 30 were classified as having proliferative DR.

### Interobserver Agreement of Perivascular Space Evaluations

The inter-rater reliabilities were good for both BG-PVS (κ = 0.76) and CSO-PVS (κ = 0.73).

### Clinical, Cerebral Small Vessel Disease, and Retinal Ganglion Cell Layer According to Diabetic Retinopathy Stage

The clinical and neuropsychological characteristics of the non-proliferative and proliferative DR groups are summarized in Table 1. There were no significant differences between the clinical or neuropsychological characteristics of these two groups.

**TABLE 1:** Clinical characteristics and neuropsychological function according to the diabetic retinopathy (DR) stage.

	Non-proliferative DR (*n* = 51)	Proliferative DR (*n* = 30)	*P* value*
**Basic clinical variables**			
Age (years)	68.7 ± 9.2	64.9 ± 10.7	0.094
Sex (female)	27 (52.9)	22 (73.3)	0.070
Year of education	9.2 ± 4.3	8.8 ± 4.2	0.250
Hypertension	20 (54.1)	11 (39.3)	0.238
Hyperlipidemia	3 (8.1)	0 (0)	0.253
Cardiovascular disease	9 (24.3)	4 (14.3)	0.316
Current or previous history of smoking	1 (2.7)	3 (10.7)	0.307
**Neuropsychological assessment**			
MMSE	23.6 ± 4.4	23.9 ± 5.6	0.863
Cognitive impairment (adjusted MMSE score < 24)	34 (67)	15 (50)	0.163
CDR	0.5 (0.5–0.5)	0.5 (0.5–0.5)	0.720
GDS	3 (3–3)	3 (3–3)	0.992
Total attention score	25.9 ± 27.3	34.6 ± 32.1	0.877
Total language score	19.5 (2.1–48.9)	30.8 (8.0–51.1)	0.725
Total visuospatial function score	42.2 (7.1–63.2)	44.3 (4.5–65.2)	0.855
Total memory score	10.9 (1.4–44.1)	27.3 (4.9–49.7)	0.544
Frontal executive function score	2.6 (0.6–46.6)	13.8 (1.2–50.3)	0.496

*Data were expressed as means with SDs or median with interquartile range or as numbers with percentages in parentheses.*

*Calculated from the chi-squared test for categorical variables, and the independent t-test or Mann–Whitney U test for continuous variables according to normality.

**FIGURE 1:**
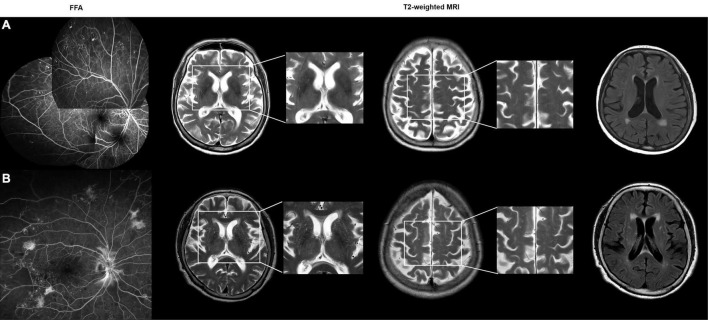
**(A)** A 83-year-old female with non-proliferative diabetic retinopathy (DR). Patchy areas of capillary dropout with microaneurysms were observed on a montage fundus fluorescein angiography (FFA) image. The axial T2-weighted MRI images show grade 0 perivascular space in the basal ganglia (BG-PVS). On fluid-attenuated inversion recovery (FLAIR) images, the total Fazekas score was 2. The total cerebral small vessel disease (SVD) score was 0. **(B)** A 73-year-old female with proliferative DR. Wide-field FFA image shows the presence of neovascularizations elsewhere around the wider areas of non-perfusion. The axial T2-weighted images show grade 4 BG-PVS. On FLAIR images, the total Fazekas score was 3. The total cerebral SVD score was 1.

**FIGURE 2:**
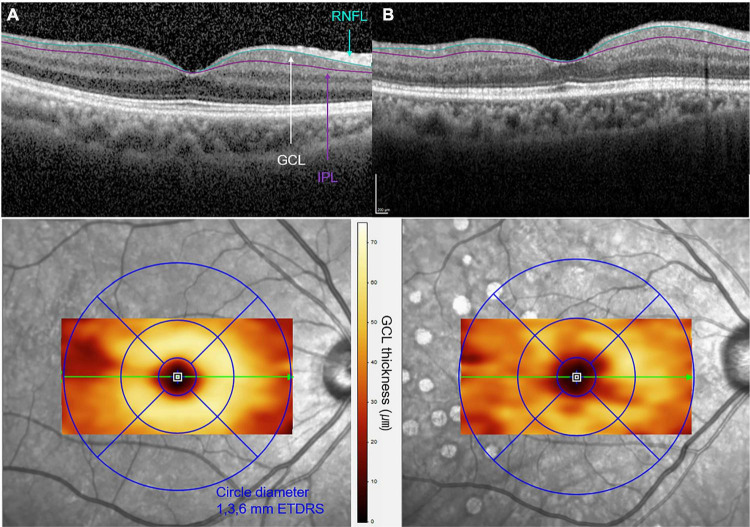
Optical coherence tomographic (OCT) images of **(A)** a 62-year-old female with non-proliferative DR and of **(B)** a 63-year-old female with proliferative DR. A cross-sectional B-scan of foveal center within the 6 × 6-mm macular cubes is shown in the upper row. The segmentation lines on the B-scan represent the inner retinal nerve fiber layer (RNFL) and outer inner plexiform layer (IPL) boundaries of the slab of ganglion cell layer (GCL). The increased irregularity of the GCL boundaries is observed in **(B)** when compared to **(A)**. En-face OCT image with color topography of each GCL thickness corresponding to the Early Treatment Diabetic Retinopathy Study (ETDRS) circles is presented in the bottom row. A decrease in GCL thickness is presented in panel B compared to panel A, as a significant color change in the 1- to 3-mm perifoveal zone.

Brain MRI and retinal OCT imaging findings for patients in the two DR groups are summarized in Table 2. Patients in the proliferative DR group had a significantly higher BG-PVS severity (*P* = 0.012) and a significantly higher total cerebral SVD score (*P* = 0.035) than patients in the non-proliferative DR group. Patients with proliferative DR also showed significantly reduced GCL thicknesses in the inferior (*P* = 0.027), superior (*P* = 0.046), and temporal (*P* = 0.038) subfields compared to patients with non-proliferative DR. Decreased GCL thicknesses were also identified in other subfields (i.e., foveal center and nasal) in the proliferative DR group; however, these results did not reach statistical significance. Figures 1, 2 show representative cases at different stages of DR progression.

**TABLE 2:** Cerebral and ophthalmic imaging findings according to the DR stage.

	Non-proliferative DR (*n* = 51)	Proliferative DR (*n* = 30)	*P* value*
**Brain MRI imaging findings**			
BG-PVS	**1 (1**–**2)**	**2 (1**–**3)**	**0.012**
CSO-PVS	2 (1–2)	2 (1–4)	0.074
Total no. of old lacunes	0 (0–0)	0 (0–1)	0.072
Total no. of microbleeds	0 (0–0)	0 (0–0)	0.763
Total WMH	3 (2–4)	3 (2–4)	0.303
Total cerebral SVD score	**1 (0**–**2)**	**1 (1**–**2)**	**0.026**
**Retinal GCL thickness (μm)**			
Foveal center	17.26 ± 6.56	16.50 ± 8.01	0.531
Inferior	**48.53 ± 10.16**	**41.26 ± 13.60**	**0.027**
Superior	**48.42 ± 10.98**	**42.69 ± 13.56**	**0.046**
Nasal	48.36 ± 8.76	44.94 ± 11.06	0.094
Temporal	**45.51 ± 10.68**	**39.35 ± 11.81**	**0.038**

*Data are expressed as means with SDs or median with interquartile range or as numbers with percentages in parentheses. A P-value in bold indicates statistical significance (P < 0.05).*

*Calculated from the chi-squared test for categorical variables, and the independent t-test or Mann–Whitney U test for continuous variables according to normality.

### Correlation of BG-PVS Severity and Total Cerebral Small Vessel Disease Score With MMSE Score

**FIGURE 3:**
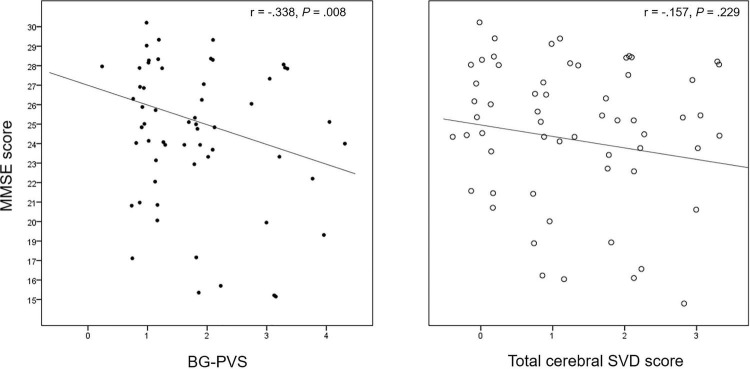
Correlation plot between the BG-PVS severity and the total cerebral SVD score with the Mini-Mental State Examination (MMSE) score.

The results of the correlation analysis for cerebral SVD makers and neuropsychological function are summarized in Supplementary Table 1. The BG-PVS severity was negatively correlated with the MMSE score (*P* = 0.007), while the total cerebral SVD score did not show a significant correlation with this score (*P* = 0.23). Figure 3 shows a corresponding scatterplot. In addition, BG-PVS was negatively correlated with total visuospatial score (*P* = 0.039), while it was positively correlated with CDR (*P* = 0.043) and GDS (*P* = 0.004). Total cerebral SVD score presented a negative correlation with frontal executive function (*P* < 0.001) and a positive correlation with GDS (*P* = 0.035).

The CSO-PVS showed a negative correlation with MMSE (*P* = 0.005). Total WMH was negatively correlated with total attention (*P* = 0.015), visuospatial (*P* = 0.023), memory (*P* = 0.025), and frontal executive (*P* < 0.001) function scores.

### Correlation of Retinal Ganglion Cell Layer Thickness With BG-PVS Severity and Total Cerebral Small Vessel Disease Score

**FIGURE 4:**
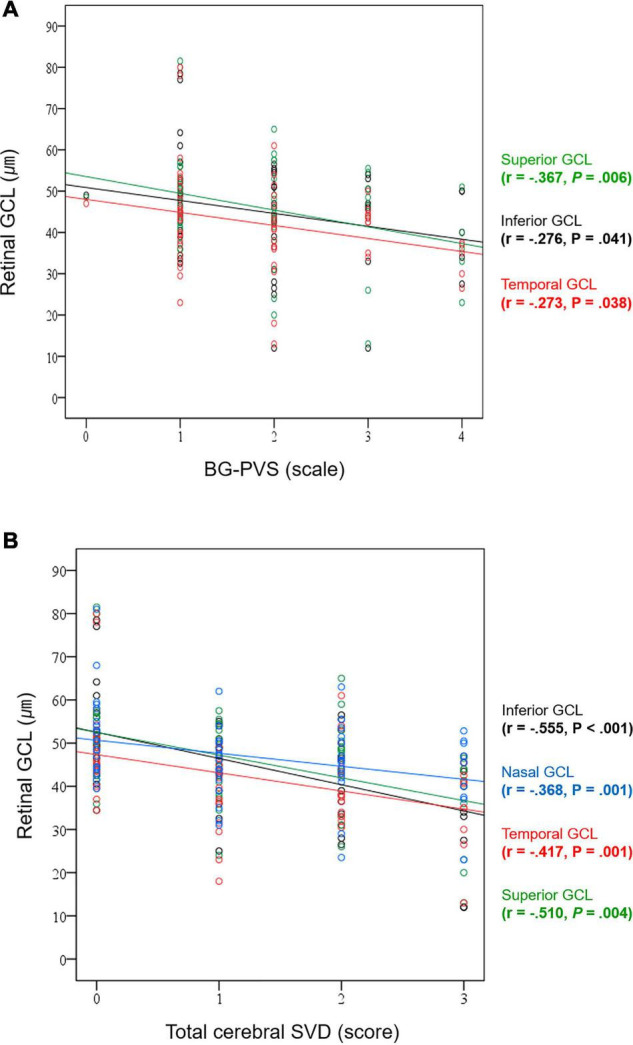
Correlation plots between **(A)** BG-PVS severity and GCL thickness and **(B)** total cerebral SVD score and GCL thickness.

The correlation analysis results between the GCL thickness and the BG-PVS severity and total cerebral SVD score are summarized in Supplementary Table 2. In this analysis, the GCL thickness showed significant negative correlations with the BG-PVS severity (*P* = 0.041 for inferior; *P* = 0.006 for superior; and *P* = 0.038 for temporal GCL, respectively) and the total cerebral SVD score (*P* < 0.001 for inferior; *P* = 0.004 for superior; and *P* = 0.001 for nasal and temporal GCL, respectively). Figure 4 shows corresponding scatterplots.

Supplementary Table 2 also summarizes the results of correlation analyses for the thickness of retinal GCL with other cerebral SVD-related markers. GCL thickness showed significant negative correlations with BG-PVS (*P* = 0.041 for inferior; *P* = 0.006 for superior; and *P* = 0.038 for temporal GCL, respectively), total number of lacunes (*P* < 0.001 for inferior and superior; and *P* = 0.012 for temporal GCL, respectively), total WMH (*P* < 0.001 for inferior; *P* = 0.005 for superior; *P* = 0.015 for nasal; and *P* = 0.042 for temporal GCL, respectively), and total CSVD (*P* < 0.001 for inferior; *P* = 0.004 for superior; and *P* = 0.001 for nasal and temporal GCL, respectively). CSO-PVS was not significantly associated with GCL thickness.

### Logistic Regression Analysis for Diabetic Retinopathy Stage Progression and Cognitive Impairment

The determinants among cerebro-ophthalmic parameters obtained by the logistic regression analysis are presented in Table 3. Possible predictors (i.e., age, male sex, underlying cardiovascular diseases, BG-PVS, CSO-PVS, total number of lacunes and WMH, total cerebral SVD score, and inferior and superior GCL thickness) that were significant in previous comparison and correlation tests were included in the multivariable logistic regression models with the enter method. According to the developed model, a higher BG-PVS score (OR = 2.306, *P* = 0.017) was associated with a higher risk of pathological angiogenesis in DR, while old age (OR = 0.138, *P* = 0.004) was associated with lower risk. Old age (OR = 2.620, *P* = 0.012) and higher BG-PVS score (OR = 1.716, *P* = 0.044) were associated with a higher risk of cognitive impairment, while thicker inferior GCL (OR = 0.669, *P* = 0.011) was associated with a lower risk.

**TABLE 3:** Multivariable logistic regression models for the determination of significant cerebro-ophthalmic parameters for DR progression (proliferative stage) and for cognitive impairment (adjusted MMSE score < 24).

Variables	DR progression		Cognitive impairment	
	OR (95% CI)	*P* Value	OR (95% CI)	*P* Value
Age > 65	**0.14 (0.04–0.54)**	**0.004**	**2.62 (0.83–8.31)**	**0.012**
Sex (male)	0.51 (0.12–2.12)	0.351	0.98 (0.27–3.59)	0.971
Underlying cardiovascular diseases	0.65 (0.17–2.54)	0.535	1.53 (0.39–6.05)	0.543
BG-PVS	**2.31 (1.16–4.59)**	**0.017**	**1.72 (1.01–2.90)**	**0.044**
CO-PVS	1.51 (0.75–3.03)	0.251	1.14 (0.43–3.02)	0.791
Total no. of old lacunes	2.64 (0.66-10.57)	0.172	1.85 (0.72–4.74)	0.199
Total WMH	0.66 (0.27–1.59)	0.354	1.83 (0.78–4.31)	0.166
Total cerebral SVD	0.63 (0.18–2.20)	0.464	0.48 (0.16–1.46)	0.193
GCL_superior	1.04 (0.94–1.16)	0.446	1.06 (0.93–1.20)	0.380
GCL_inferior	0.93 (0.83–1.044)	0.223	**0.67 (0.30–1.04)**	**0.011**

*A P-value in bold indicates statistical significance (P < 0.05).*

## Discussion

In this study, we assessed the relationships between cerebral SVD severity and retinal GCL parameters based on the DR stage. Higher BG-PVS severity, higher total cerebral SVD score, and reduced retinal GCL thicknesses in the inferior, superior, and temporal subfields were identified in the proliferative DR group compared with the non-proliferative DR group. In addition, the BG-PVS severity was negatively correlated with the MMSE score, while the retinal GCL thickness was negatively correlated with the BG-PVS severity and the total cerebral SVD score. Taken together, these findings suggest that the BG-PVS severity reflects the retinal neurodegeneration that precedes pathological angiogenesis during DR progression. Moreover, the BG-PVS severity may be a potential biomarker of cognitive decline in patients with DR. Finally, our results provide possible clinical evidence of a link between the retina and the brain on the cognitive impairment in patients with DR.

While a relationship between enlarged PVS and cerebral SVD has been noted (Doubal et al., 2010), PVS have also been proposed to be a part of a macroscopic clearance mechanism called the glymphatic system (Jessen et al., 2015), which may be related to the development of neurodegenerative diseases. A previous study has shown that T2DM suppresses the clearance of interstitial fluid, leading to the impairment of glymphatic system (Jiang et al., 2017). Although the mechanism underlying enlarged PVS is not completely understood, previous studies have shown that associations of disease with visible PVS differ based on their location (Charidimou et al., 2017; Chen et al., 2019). The BG-PVS burden has been associated with hypertensive angiopathy, systemic markers of inflammation, lacunar stroke, and vascular cognitive impairment (Hansen et al., 2015; Chen et al., 2019), while the CSO-PVS burden has been associated with cerebral amyloid angiopathy and Alzheimer’s disease (van Veluw et al., 2016; Chen et al., 2019; Sepehrband et al., 2021). In our study, the BG-PVS severity was associated with a higher DR grade, suggesting that pathological angiogenesis in DR is associated with the systemic markers of inflammation and vascular cognitive impairment. Although a previous meta-analysis has shown that enlarged PVS is not associated with T2DM (Francis et al., 2019), our discrepant results may have been related to the fact that we assessed enlarged PVS based on the DR stage, which represents the diabetic neurovascular remodeling process. Proliferative DR is present only in 10–12% of patients with T2DM (Thomas et al., 2015), and previous studies have not evaluated whether the PVS burden is increased with higher DR severity. Our results provide a better understanding of the pathogenesis underlying brain manifestations during the progression of DR.

Previous studies have shown an increased burden of lacunes in patients with T2DM and DR (Inoue et al., 1998; Sanahuja et al., 2016). The relationship between diabetes and microbleeds, however, remains unclear (Umemura et al., 2017), and the observational studies have reported discordant results concerning the relationship between diabetes and WMH (van Elderen et al., 2010; Sanahuja et al., 2016; de Bresser et al., 2018). In our study, these cerebral SVD markers did not show significant differences based on DR grade. The total cerebral SVD score has recently been proposed as a potential scoring system for the DR stage and has been shown to better represent the overall effects of SVD on the brain than one or two features independently (Cuadrado-Godia et al., 2018). Our results also suggest that the total cerebral SVD score may well reflect the DR stage.

Furthermore, our results suggest a potential effect of BG-PVS severity on the early stages of cognitive impairment in patients with DR. Recent studies have identified an association between BG-PVS severity and cognitive impairment in patients with Parkinson’s disease (PD) and stroke (Arba et al., 2018; Park et al., 2019). These findings suggest that the BG-PVS severity may be a novel indicator of the early pathophysiological processes leading to cognitive impairment in the brain. Considering a recent study showing that PVS from other brain regions may also be associated with PD (Donahue et al., 2021), future studies should consider various anatomical regions for PVS evaluation.

Similar to the glymphatic system of the brain, the existence of neurovascular units in the retina has also been proposed (Metea and Newman, 2007; Newman, 2013). Blood-retinal barrier disruption and retinal neurodegeneration (Bek, 2017; Duh et al., 2017) are more significantly activated by low-grade inflammation (Du et al., 2013; Altmann and Schmidt, 2018) and are early events during the pathogenesis of DR. Glial activation and neuronal apoptosis, both being the prominent features of retinal neurodegeneration (Simó and Hernández, 2014), result in decreased retinal GCL thickness. An association between a reduced retinal GCL thickness and the SVD burden in the brain was first identified in this study through a correlation analysis between the BG-PVS severity and the total cerebral SVD score. These findings suggest that DR progression and cognitive impairment in diabetic patients may share specific mechanisms mainly related to systemic microangiopathy. This common mechanism appears to be associated with the changes in GCL thickness and BG-PVS severity. Further longitudinal studies are necessary to validate the sequence of these changes.

It should be noted that there were several limitations to our study. First, this study was based on the retrospective, single-institutional data with a relatively small size without complete control of covariates and external validation potentially limiting the generalizability of our results. However, to minimize the selection bias, we ensured that the basic characteristics such as age and sex and the underlying conditions of the subgroups were comparable. Second, the lack of an age-matched control group without DR prevents the extrapolation of these results to the general diabetic population. Future studies with the inclusion of age-matched control groups are warranted. Third, variables such as brain volume or body mass index, which have shown an association with PVS (Barisano et al., 2021; Liu et al., 2021), were not analyzed in this study. Further studies should also include these variables in a larger dataset.

## Conclusion

Our results demonstrate that the BG-PVS severity may be a novel imaging biomarker in patients with DR, reflecting progression in the DR stage and cognitive impairment. Furthermore, our identification of a possible association between a decreased GCL thickness and the BG-PVS severity provides potential clinical evidence of a link between retinal and brain changes during diabetic neurodegeneration.

## Data Availability Statement

The corresponding author had full access to all the data in the study and takes responsibility for the integrity of the data and the accuracy of the data analysis. The raw data supporting the conclusions of this article will be made available by the correspondence, without undue reservation.

## Ethics Statement

The studies involving human participants were reviewed and approved by the Severance Institutional Review Board (IRB approval number: 2020-3812-001). Written informed consent for participation was not required for this study in accordance with the National Legislation and the Institutional Requirements.

## Author Contributions

EYC and YWP contributed to the concept, design, and drafting of the manuscript and performed the statistical analysis. EYC, YWP, MK, CSL, SSA, JK, and S-KL contributed to the acquisition, analysis, and interpretation of data. EYC, YWP, and ML contributed to the critical revision of the manuscript for important intellectual content, administrative, technical, material support, and supervision. YWP obtained funding. All authors contributed to the article and approved the submitted version.

## Conflict of Interest

The authors declare that the research was conducted in the absence of any commercial or financial relationships that could be construed as a potential conflict of interest.

## Publisher’s Note

All claims expressed in this article are solely those of the authors and do not necessarily represent those of their affiliated organizations, or those of the publisher, the editors and the reviewers. Any product that may be evaluated in this article, or claim that may be made by its manufacturer, is not guaranteed or endorsed by the publisher.

## Abbreviations

BG: basal ganglia
CDR: clinical dementia rating scale
CSO: centrum semiovale
CSVD: cerebral small vessel disease
DR: diabetic retinopathy
FOV: field of view
FLAIR: fluid-attenuated inversion recovery
GCL: ganglion cell layer
GDS: global deterioration scale
MMSE: Mini-Mental State Examination
OCT: optical coherence tomography
OR: odds ratio
PVS: perivascular space
SNSB: Seoul neuropsychological screening battery
SVD: small vessel disease
TE: echo time
TR: repetition time
T2DM: type 2 diabetes mellitus
WMH: white matter hyperintensity.
