# Supplementary material for: Magnetic Resonance Imaging-Visible Perivascular Spaces in the Basal Ganglia Are Associated With the Diabetic Retinopathy Stage and Cognitive Decline in Patients With Type 2 Diabetes
Source: Front Aging Neurosci. 2021 Nov 12;13:666495. doi: 10.3389/fnagi.2021.666495 (PMC8633948; doi:10.3389/fnagi.2021.666495)
Supplement: Supplementary file 1 [file Data_Sheet_1.docx]

**Supplemental Table 1.** Association between neuropsychological function (MMSE, CDR, GDS, and SNSB) and cerebral SVD markers.

| **r (*P* Value)** | | **Neuropsychological function** | | | | | | | |
| --- | --- | --- | --- | --- | --- | --- | --- | --- | --- |
|  |  | **Attention** | **Language** | **Visuospatial** | **Memory** | **Frontal** | **CDR** | **GDS** | **MMSE** |
| **Brain SVD markers** | **BG-PVS** | -.237  (.126) | -.078  (.620) | **-.316**  **(.039)** | -.091  (.562) | -.284  (.065) | **.299**  **(.043)** | **.385**  **(.004)** | **-.346**  **(.007)** |
|  | **CSO-PVS** | -.085  (.586) | .058  (.710) | -.252  (.103) | -.063  (.687) | -.157  (.313) | .289  (.052) | .263  (.052) | **-.358**  **(.005)** |
|  | **Total no. of old lacunes** | -.005  (.975) | -.098  (.533) | -.237  (.125) | -.135  (.389) | -.236  (.127) | .017  (.912) | .166  (.226) | -.032  (.810) |
|  | **Total no. of microbleeds** | -.098  (.531) | -.111  (.478) | .004  (.978) | -.071  (.652) | -.214  (.168) | -.069  (.650) | -.092  (.505) | .055  (.682) |
|  | **Total WMH** | **-.368**  **(.015)** | -.223  (.151) | **-.346**  **(.023)** | **-.342**  **(.025)** | **-.549**  **(<.001)** | .106  (.481) | .264  (.051) | -.176  (.183) |
|  | **Total cerebral SVD score** | -.250  (.106) | -.171  (.274) | -.298  (.053) | -.268  (.083) | **-.576**  **(<.001)** | .052  (.731) | **.285**  **(.035)** | -.156  (.237) |

Coefficient of correlation (r) was derived from Pearson’s correlation test. A P-value in bold indicates statistical significance (P < .05).

BG = basal ganglia; CSO = centrum semiovale; PVS = perivascular space; WMH = white matter hyperintensity; SVD = small vessel disease; CDR = Clinical Dementia Rating Scale; CDR-SOB = CDR-sum of boxes; GDS = Global Deterioration Scale; MMSE = Mini-Mental State Examination

**Supplemental Table 2.** Association between the thickness of retinal GCL and various cerebral SVD markers.

| **r (*P* Value)** | | **Retinal GCL thickness** | | | | |
| --- | --- | --- | --- | --- | --- | --- |
|  |  | **Foveal center** | **Inferior** | **Superior** | **Nasal** | **Temporal** |
| **Brain SVD markers** | **BG-PVS** | .052  (.703) | **-.276**  **(.041)** | **-.367**  **(.006)** | -.194  (.145) | **-.273**  **(.038)** |
|  | **CSO-PVS** | .068  (.614) | -.228  (.094) | -.260  (.055) | -.104  (.437) | -.198  (.136) |
|  | **Total no. of old lacunes** | -.003  (.980) | **-.468**  **(<.001)** | **-.457**  **(<.001)** | -.202  (.129) | **-.329**  **(.012)** |
|  | **Total no. of microbleeds** | -.111  (.411) | -.129  (.346) | -.132  (.336) | -.224  (.092) | -.166  (.212) |
|  | **Total WMH** | .004  (.979) | **-.469**  **(<.001)** | **-.371**  **(.005)** | **-.318**  **(.015)** | **-.268**  **(.042)** |
|  | **Total cerebral SVD score** | -.062  (.647) | **-.555**  **(<.001)** | **-.510**  **(.004)** | **-.368**  **(.001)** | **-.417**  **(.001)** |

Coefficient of correlation (r) was derived from Pearson’s correlation test. A P-value in bold indicates statistical significance (P < .05).

BG = basal ganglia; CSO = centrum semiovale; PVS = perivascular space; WMH = white matter hyperintensity; SVD = small vessel disease; RNFL = retinal nerve fiber layer; GCL = ganglion cell layer
